# A novel technology for disinfecting surfaces infested with Candida auris: the UVC chip

**DOI:** 10.1093/eurpub/ckac131.251

**Published:** 2022-10-25

**Authors:** E Frongillo, D Amodeo, N Nante, G Cevenini, G Messina

**Affiliations:** 1 Post Graduate School of Public Health, University, Siena, Italy; 2 Department of Medical Biotechnologies, University, Siena, Italy; 3 Department of Molecular and Developmental Medicine, University, Siena, Italy

## Abstract

**Background:**

The fungal pathogen Candida Auris is increasingly associated with multidrug-resistant infections that are highly expensive for the Health Care System. The spreading of this pathogen can occur, among others, through contact with infected surfaces or medical instruments. This study evaluated the efficacy of a novel UVC chip, novel alternative to UVC LEDs and lamps, in inactivating Candida auris strain.

**Methods:**

This experimental study was carried out between July and September 2020 at the University of Siena. Candida auris (ATCC 12372) at two known concentrations (1.5X107 and 1.5x106 CFU/ml) at a fixed distance (7,5 cm) from the chip (5.1mW radiant power) was tested, in triplicates, with three exposure times (5, 10 and 15 minutes). Potato Dextrose Agar (PDA) plates without the plate lid and containing Candida auris were exposed to UVC light. Subsequently, the plates were incubated at 36 °C for 48 h. Log reduction between treated and positive control (not exposed to UVC light) samples was calculated.

**Results:**

At 15 minutes, we had the highest inactivation result, mean 4.43 log10, starting from a 1.5x106 CFU/mL concentration. At a higher concentration, 1.5X107 CFU/mL, the reduction had a mean of 3.51 log10.

**Conclusions:**

The results of the experiments showed a significant microbial reduction in relation to the exposure time. The highest level of reduction was reached after 15 minutes of exposure. UVC chip had a relevant biocidal effect on Candida auris and may represent a valuable tool in the prevention of infections caused by this pathogen, which is becoming increasingly prevalent and persistent globally.

**Key messages:**

• The use of UVC Chip decreases surface contamination.

• New technology against healthcare-associated infections.

